# Maternal Nutrient Restriction Disrupts Gene Expression and Metabolites Associated with Urea Cycle, Steroid Synthesis, Glucose Homeostasis, and Glucuronidation in Fetal Calf Liver

**DOI:** 10.3390/metabo12030203

**Published:** 2022-02-24

**Authors:** Susumu Muroya, Yi Zhang, Kounosuke Otomaru, Kazunaga Oshima, Ichiro Oshima, Mitsue Sano, Sanggun Roh, Koichi Ojima, Takafumi Gotoh

**Affiliations:** 1 Division of Animal Products Research, NARO Institute of Livestock and Grassland Science (NILGS), Ibaraki, Tsukuba 305-0901, Japan; koojima@affrc.go.jp; 2 Department of Agricultural Sciences and Natural Resources, Kagoshima University, Korimoto 1-21-24, Kagoshima 890-8580, Japan; zhangyi439250@gmail.com (Y.Z.); oshima@agri.kagoshima-u.ac.jp (I.O.); 3 Joint Faculty of Veterinary Medicine, Kagoshima University, Korimoto 1-21-24, Kagoshima 890-8580, Japan; otomaru@vet.kagoshima-u.ac.jp; 4 Division of Year-Round Grazing Research, NARO Western Region Agricultural Research Center, 60 Yoshinaga, Ohda 694-0013, Shimane, Japan; tenpoint@affrc.go.jp; 5 Faculty of Human Culture, University of Shiga Prefecture, 2500 Hassaka-cho, Hikone 522-8533, Shiga, Japan; sano.m@shc.usp.ac.jp; 6 Graduate School of Agricultural Science, Tohoku University, 468-1 Aoba, Aramaki, Aoba-ku, Sendai 980-8578, Miyagi, Japan; sanggun.roh@tohoku.ac.jp

**Keywords:** fetal programming, glucuronidation, fetal growth restriction (fgr), liver, maternal nutrient restriction, steroid synthesis, urea cycle

## Abstract

This study aimed to understand the mechanisms underlying the effects of maternal undernutrition (MUN) on liver growth and metabolism in Japanese Black fetal calves (8.5 months in utero) using an approach that integrates metabolomics and transcriptomics. Dams were fed 60% (low-nutrition; LN) or 120% (high-nutrition; HN) of their overall nutritional requirements during gestation. We found that MUN markedly decreased the body and liver weights of the fetuses; metabolomic analysis revealed that aspartate, glycerol, alanine, gluconate 6-phosphate, and ophthalmate levels were decreased, whereas UDP-glucose, UDP-glucuronate, octanoate, and 2-hydroxybutyrate levels were decreased in the LN fetal liver (*p* ≤ 0.05). According to metabolite set enrichment analysis, the highly different metabolites were associated with metabolisms including the arginine and proline metabolism, nucleotide and sugar metabolism, propanoate metabolism, glutamate metabolism, porphyrin metabolism, and urea cycle. Transcriptomic and qPCR analyses revealed that MUN upregulated *QRFPR* and downregulated genes associated with the glucose homeostasis (*G6PC*, *PCK1*, *DPP4*), ketogenesis (*HMGCS2*), glucuronidation (*UGT1A1*, *UGT1A6*, *UGT2A1*), lipid metabolism (*ANGPTL4*, *APOA5*, *FADS2*), cholesterol and steroid homeostasis (*FDPS*, *HSD11B1*, *HSD17B6*), and urea cycle (*CPS1*, *ASS1*, *ASL*, *ARG2*). These metabolic pathways were extracted as relevant terms in subsequent gene ontology/pathway analyses. Collectively, these results indicate that the citrate cycle was maintained at the expense of activities of the energy metabolism, glucuronidation, steroid hormone homeostasis, and urea cycle in the liver of MUN fetuses.

## 1. Introduction

Maternal undernutrition (MUN) during gestation in mammals causes retardation of fetal development, which is a typical fetal growth restriction (FGR; also known as intrauterine growth restriction) [1]. Low levels of calories, proteins, fats, and/or micronutrients in pregnant maternal diets impair the growth and metabolism of fetal organs in mammals, including humans [1]. MUN results in low birth weight and restricted growth of organs such as the brain, heart, skeletal muscle, liver, thymus, and kidney in rats [2] and sheep [3]. Even if there is no phenotypic impact, gene expression and metabolites in fetal organs are altered due to physiological adaptations in response to low nutrient levels, which are linked to epigenetic changes, including DNA methylation in mise and rats [4]. This epigenetic mechanism is thought to promote the development and growth of MUN fetal organs to establish a thrifty constitution in nutrient-restricted dams during gestation in mammals [5]. MUN predisposes the fetus to metabolic disorders, which cause prolonged disrupted homeostasis in the offspring, such as insulin resistance. Metabolic disruption can lead to obesity, type II diabetes, hypertension, apoplexy, and cardiac infarction in the adulthood of MUN offspring [5]. Although MUN affects fetuses and offspring of nutrient-restricted dams differently, the impact of MUN on FGR depends on the animal species, gestational stage, extent and period of restriction, and types of restricted nutrients such as protein and calories [6]. In livestock production, MUN causes huge economic losses to farm animal producers. It is necessary to avoid MUN, especially in meat animal production, by appropriate management of dams with careful monitoring. In addition, farm animals such as sheep are good models to investigate the impact of MUN on human fetuses and offspring due to the high similarity to human placental and fetal development compared to rodents [7], especially in terms of the developmental origin of metabolic disease in adulthood. 

The liver is one of the organs most susceptible to MUN [2]. Disruption of the liver has an adverse impact on the entire body owing to its regulatory role in the systemic metabolism of energy, nutrients, and xenobiotics for homeostasis and physiological adaptation. The liver growth and metabolism of fetuses in global nutrient/calorie-restricted dams are impaired in rats, guinea pigs, and sheep [8,9,10,11]. Based on previous analyses of gene expression and metabolite content, metabolism of energy substrates, including glucose and fatty acids, and the networks of metabolic regulatory factors/receptors, such as insulin-like growth factors (IGFs), leptin, and glucocorticoids, are disrupted in fetuses or offspring of calorie-restricted mice [12], global nutrient-restricted sheep [10,13], protein-restricted pigs [14], and global nutrient- and protein-restricted cattle [15,16]. Elevated oxidative stress and reduced antioxidant activity have also been observed in sheep fetuses of calorie-restricted [11] and global nutrient-restricted dams [17]. In addition, gene expression associated with cholesterol biosynthesis, an essential metabolic process for hormone synthesis in the liver, was reduced in the fetal or offspring liver of protein-restricted rats [18], global nutrient-restricted rats [19], and sheep [20]. Moreover, the phenotypes of the offspring from global nutrient-restricted dams have shown that MUN adversely programmed the metabolism of the fetal liver in utero, and the programmed effect lasted for the long term in sheep, even in adulthood [10,13]. Metabolic disorders in the liver of offspring could also have negative effects on other organs through a disrupted regulatory role in systemic homeostasis. However, the impact of MUN on liver metabolism in fetuses and offspring is poorly understood, especially the genes/metabolism that are most affected by MUN in cattle. 

Recently, we demonstrated that amino acid (AA) metabolism and gene expression associated with energy metabolism (apelin receptor, carnitine palmitoyltransferase 1B, uncoupling protein 2, etc.), glucose homeostasis (enolase 3, glucose-6-phosphate isomerase, fructose bisphosphatase 2, etc.), and angiogenesis (angiopoietin-like 4, NO synthetase 2, NO synthetase 3) were decreased in the *longissimus* muscle of fetal calves from global nutrient-restricted dams during the entire gestation period compared with unrestricted fetuses [21]. This suggests that insufficient energy in MUN fetuses reduced the use of AAs for protein synthesis at the expense of skeletal muscle growth and development. In MUN fetal calves, skeletal muscle development was disrupted based on the analyses of histochemistry and gene expression associated with myogenic regulatory factors (under review). In MUN fetal carcasses, pronounced growth restriction of the liver, kidney, thymus, spleen, heart, lung, rumen, omasum, and large intestine has been observed, as well as restriction of 12 skeletal muscles [22]. Given that glucose and fatty acid metabolism were disrupted in the fetal muscle of low nutrition (LN) dams, we hypothesized that the function of the liver, the central regulator of systemic energy and hormone metabolism, could also be disrupted in response to insufficient energy substrates from dams to the placenta. 

In the present study, we aim to elucidate the effects of MUN on fetal liver metabolism in Japanese Black (JB) cattle. To address this, we used a design in which pregnant dams are fed on low nutrient (LN) and high nutrient (HN) diets (based on protein, fat, and energy contents) during the entire gestation (until month 8.5 post-conception). The LN and HN treatments were set to 60% and 120% of the recommended nutrient level, respectively, for the pre-pregnant body weight (BW) of JB cattle as was in the previous studies [21,22]. We analyzed changes in the metabolome and transcriptome in the fetal liver in an integrative approach using capillary electrophoresis time-of-flight mass spectrometry (CE-TOFMS) and microarray analysis. Subsequently, we performed bioinformatic analysis of the metabolomic and transcriptomic data to understand the impact of MUN on fetal liver metabolism.

## 2. Results

### 2.1. Fetal Carcass Traits

First, we investigated the effect of MUN on the growth of the whole body and liver of fetal calves. The BW and liver weight of the LN group were lower than those of the HN group (*p* ≤ 0.05), whereas the percentage of liver weight in the BW group did not differ (*p* > 0.05) (Table 1). The ratios of BW and liver in the LN group to those in the HN group were 0.72 and 0.78, respectively. Thus, MUN markedly decreased the mass of liver in fetuses of the LN group [22]. 

### 2.2. Metabolomic Profile of Fetal Liver

To assess the effect of MUN on fetal liver metabolism, metabolomic differences were analyzed using CE-TOFMS (Table 2). In the CE-TOFMS analysis, four fetuses with the highest BW in the HN group and four with the lowest BW in the LN group were used for the analysis. In total, 278 peaks detected by MS were annotated as metabolites (Table S1). Among these metabolites, the levels of aspartate, betaine aldehyde (BTL), glycerol, 3-aminopropane-1,2-diol (3-APRP-1,2-diol), alanine, 6-phosphogluconate (6-PG), ophthalmate (*p* ≤ 0.05), *N*^6^,*N*^6^,*N*^6^-trimethyllysine, and γ-Glu-Ser (*p* ≤ 0.10) were higher in the fetal livers of the LN group than in those of the HN group (Table 2). The levels of 4-amino-3-hydroxybutyrate (4-Amino-3-HBA), 2-aminoethylphosphonate (2-AEP, also known as ciliatine), uridine 5′-diphosphate (UDP)-glucuronate, UDP-glucose (UDP-Glc)/UDP-galactose (UDP-Gal), *N*^5^-ethylglutmine, 2-hydroxybutyrate (2-HBA), octanoate, Gly-Leu (*p* ≤ 0.05), methionine sulfoxide, and gluconate (*p* ≤ 0.10) were lower in the LN fetal liver than in the HN fetal liver.

**Figure 1:**
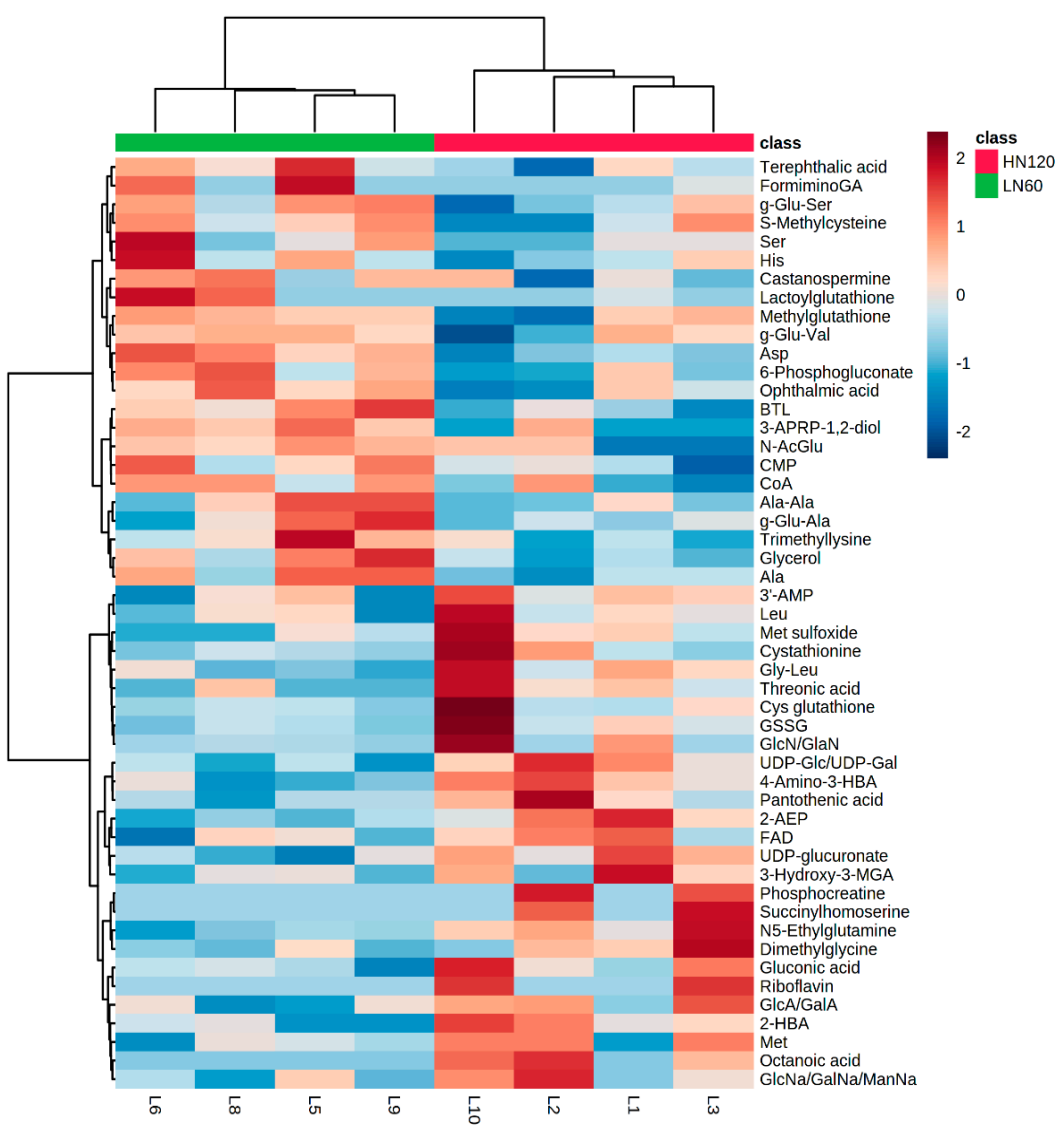
Heatmap of HCA using the top 50 statistically different liver metabolites between LN (green) and HN (red) fetuses. The row displays the metabolite, and the column represents the sample. Metabolites with relatively low contents are displayed in blue, while metabolites with relatively high contents are displayed in right brown. The brightness of each color corresponds to the magnitude of the difference when compared with the average value. FormiminoGA; formiminoglutamic acid, BTL; betaine aldehyde, 3-APRP-1,2-diol; 3-aminopropane-1,2-diol, N-AcGlu; *N*-acetyl glutamate, CMP; cytidine monophosphate, CoA; coenzyme A, 3′-AMP; 3′-adenosine monophosphate, GSSG; oxidized glutathione, GlcN/GalN; *N*-acetylglucosamine/*N*-acetylgalactosamine, GlcA/GalA; glucosamine/galactosamine, 2-HBA; 2-hydroxybutyrate, GlcNa/GalNa/ManNa; *N*-acetylglucosamine/*N*-acetylgalactosamine/*N*-acetylmannosamine, UDP-glucuronate; uridine 5′-diphosphate-glucuronate, 4-amino-3-HBA; 4-amino-3-hydroxybutyrate, 2-AEP; 2-aminoethylphosphonate, FAD; flavin adenine dinucleotide, 3-hydroxy-3-MGA; 3-hydroxy-3-methylglutarate, UDP-Glc/UDP-Gal; UDP-glucose/UDP-galactose. Amino acids and peptides are shown in three letter code.

The fetal liver samples were classified into LN and HN groups by unsupervised hierarchical clustering analysis (HCA) using the top 50 significantly different metabolites between the LN and HN groups. Figure 1 shows that aspartate, BTL, glycerol, 3-APRP-1,2-diol, alanine, 6-PG, ophthalmate, and coenzyme A (CoA) were more abundant in the LN group, whereas 2-AEP, UDP-Glc/UDP-Gal, *N*^5^-ethylglutamine, UDP-glucuronate, 2-HBA, octanoate, gluconate, pantothenate, flavin adenine dinucleotide (FAD), 3-hydroxy-3-methylglutarate (3-HMGA), 4-amino-3-HBA, cystathionine, dimethylglycine, and succinylhomoserine were more abundant in the HN group. The HCA result indicates that these metabolites highly contribute to the discrimination of the fetal liver samples between the two nutrient levels. Thus, the fetal liver of the LN group was characterized by abundant metabolites (aspartate, BTL, glycerol, alanine, 6-PG, ophthalmate, CoA) and less abundant metabolites (2-AEP, UDP-Glc/UDP-Gal, UDP-glucuronate, gluconate, pantothenate, FAD, and 3-HMGA). 

To understand biological processes associated with differentially expressed metabolites between the LN and HN groups, this study performed metabolite set enrichment analysis (MSEA) of the top 50 differentially expressed metabolites. The differentially expressed metabolites were significantly associated with the metabolic pathways of the urea cycle, malate-aspartate shuttle, AAs (arginine, proline, glutamate, tyrosine, aspartate), starch and sucrose, nucleotide sugars, propanoate, β-alanine, purine, porphyrin, betaine, and glycerolipid (*p* ≤ 0.01) (Table 3). The metabolic pathways associated with the Warburg effect, pantothenate and CoA biosynthesis, lactose synthesis, sphingolipids, androgens, and estrogen (*p* ≤ 0.05) were also significantly extracted (Table 3).

### 2.3. Effect of MUN on the Gene Expression Profile of the Fetal Liver

**Figure 2:**
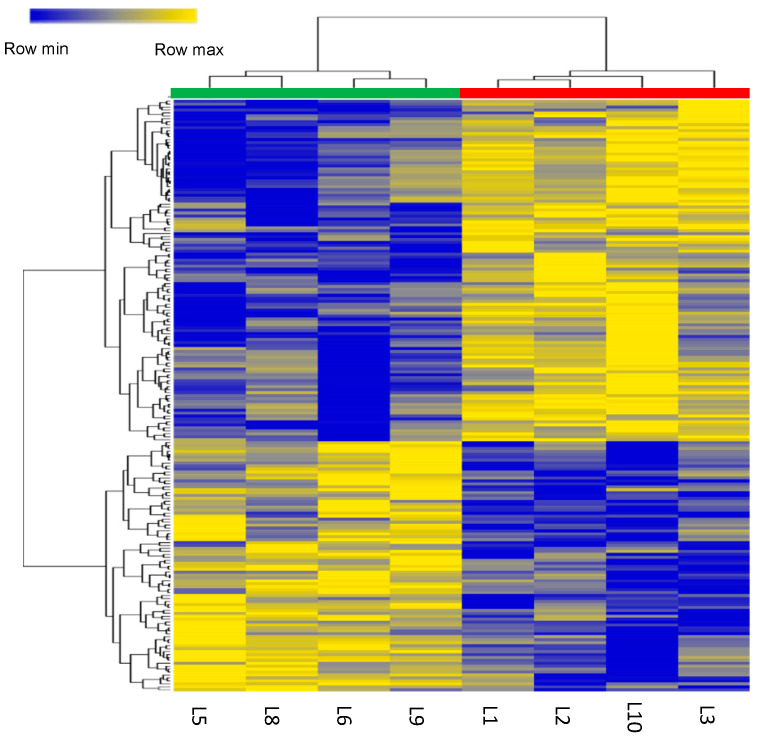
Heatmap of HCA using 201 differentially expressed genes (*p* ≤ 0.05) between the LN (green) and HN (red) fetal livers. The row displays the gene and the column represents the sample. Genes with relatively low contents are displayed in dark blue, while genes with relatively high contents are displayed in yellow. The brightness of each color corresponds to the magnitude of the difference when compared with the average value.

MUN-mediated metabolic alterations in the LN fetal liver may result from disrupted gene expression. To investigate the association between MUN-induced metabolic alterations and gene expression, a microarray-based gene expression analysis was performed using liver RNA samples. RNA was extracted from four fetuses with the highest BW in the HN group and four fetuses with the lowest BW in the LN group. Of the 12,568 unique genes detected, the expression levels of 132 genes in the fetal liver varied > two-fold between the LN and HN groups (63 upregulated and 69 downregulated genes in the LN group). The fetal liver samples were classified into LN and HN groups by HCA using the 237 microarray probe signals of differentially expressed genes (201 unique genes, *p* ≤ 0.05) between the LN and HN groups (Figure 2).

**Figure 3:**
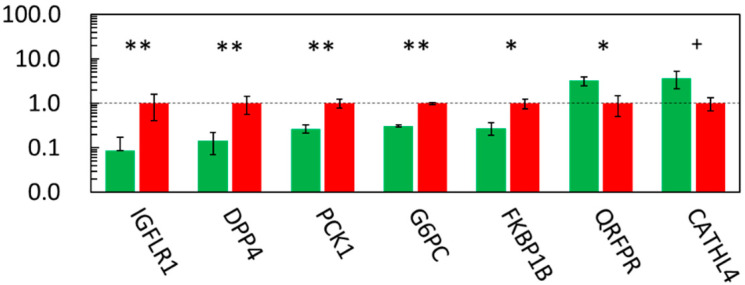
qPCR results of highly down- and upregulated fetal liver genes in microarray analysis. The ratios of the normalized gene expression of the LN (green) to HN (red) fetuses are shown as columns. Ribosomal protein lateral stalk subunit P0 (*RPLP0*) was used as the internal control. Error bars indicate SEM. **, *, and + indicate differences between the LN and HN fetuses at *p* ≤ 0.01, ≤0.05, and ≤0.10, respectively. *IGFLR1*; IGF like family receptor 1, *DPP4*; dipeptidyl-peptidase 4, *PCK1*; phosphoenolpyruvate carboxykinase 1, *G6PC*; glucose-6-phosphatase, catalytic subunit, *FKBP1B*; FK506 binding protein 1B, *QRFPR*; pyroglutamylated RFamide peptide receptor, *CATHL4*; cathelicidin 4.

The top five downregulated genes in the LN group were glucose-6-phosphatase, catalytic subunit (*G6PC*), phosphoenolpyruvate carboxykinase 1 (*PCK1*, cytoplasmic *PEPCK*), dipeptidyl-peptidase 4 (*DPP4*), FK506 binding protein 1B (*FKBP1B*), and IGF like family receptor 1 (*IGFLR1*). The top five upregulated genes in the LN group were necessary for RNA interference domain-containing (*NRDE2*), pyroglutamylated RFamide peptide receptor (*QRFPR*), proteoglycan 3 (*LOC520402*), hemoglobin subunit mu (*HBM*), and cathelicidin 4 (*CATHL4*). The downregulation of *G6PC*, *PCK1*, *DPP4*, *FKBP1B*, and *IGFLR1* (*p* ≤ 0.05) and the upregulation of *QRFPR* (*p* = 0.019) and *CATHL4* (*p* = 0.079) in the fetuses of the LN group were validated using qRT-PCR (Figure 3). 

**Figure 4:**
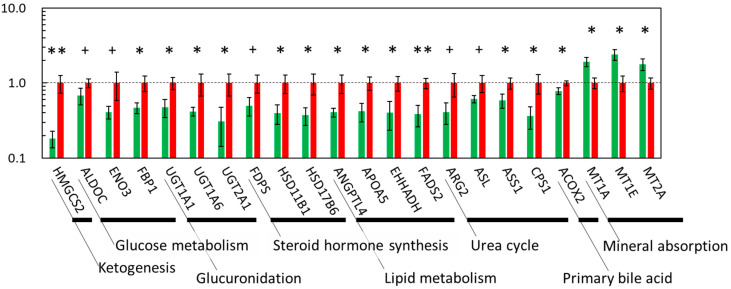
qPCR results of down- and upregulated fetal liver genes associated with essential liver metabolisms. The ratios of the normalized gene expression of the LN (green) to HN (red) fetuses are shown as columns. *RPLP0* was used as the internal control. Error bars indicate SEM. **, *, and + indicate differences between the LN and HN fetuses at *p* ≤ 0.01, ≤0.05, and ≤0.10, respectively. *HMGCS2*; 3-hydroxy-3-methylglutaryl-CoA synthase 2, *ALDOC*; aldolase, fructose-bisphosphate C, *ENO3*; enolase 3, *FBP1*; fructose-bisphosphatase 1, *UGT1A1*/*UGT1A6*/*UGT2A1*; UDP glucuronosyltransferase family-1 member A1 complex locus/-1 member A6 complex locus/-2 member A1 complex locus, *FDPS*; farnesyl diphosphate synthase, *HSD11B1*; hydroxysteroid (11-β) dehydrogenase 1, *HSD17B6*; hydroxysteroid (17-β) dehydrogenase 6, *ANGPTL4*; angiopoietin like 4, *APOA5*; apolipoprotein A-V, *EHHADH*; enoyl-CoA, hydratase/3-hydroxyacyl CoA dehydrogenase, *FADS2*; fatty acid desaturase 2, *ARG2*; arginase 2, *ASL*; argininosuccinate lyase, *ASS1*; argininosuccinate synthase 1, *CPS1*; carbamoyl-phosphate synthase 1, *ACOX2*; acyl-CoA oxidase 2, branched chain, *MT1A*/*MT1E*/*MT2A*; metallothionein-1A/-1E/-2A.

The microarray analysis results suggested that MUN affected the expression of genes associated with glucose homeostasis (*G6PC*, *PCK1*, and *QRFPR*) and fatty acid metabolism (*DPP4*) in the fetal liver of the LN group. Moreover, MSEA revealed that MUN altered the metabolic pathways associated with the urea cycle, AA metabolism, nucleic acid metabolism, and steroid hormone metabolism, which are specific to the roles of hepatocytes. Therefore, this study focused on the genes related to liver metabolism (Figure 4). 

The qPCR results showed that the expression levels of *HMGCS2*, *FADS2* (*p* ≤ 0.01), *FBP1*, *UGT1A1*, *UGT1A6*, *UGT2A1*, *HSD17B6*, *HSD11B1*, *ANGPTL4*, *APOA5*, *EHHADH*, *ASS1*, *CPS1* (*p* ≤ 0.05), *ENO3*, *ALDOC*, *FDPS*, *ARG2*, and *ASL* (*p* ≤ 0.10) were downregulated in the LN fetal liver. In contrast, the expression of *MT1A*, *MT1E*, and *MT2A* was upregulated in the LN fetal liver (*p* ≤ 0.05). Thus, these data indicate that MUN affects the expression of fetal liver genes associated with ketogenesis (*HMGCS2*), glycolysis (*ALDOC*, *ENO3*), gluconeogenesis (*G6PC*, *PCK1*, *FBP1*), glucuronidation (*UGT1A1*, *UGT1A6*, *UGT2A1*), cholesterol synthesis (*FDPS*, *HSD11B1*, *HSD17B6*), lipid metabolism (*ANGPTL4*, *APOA5*, *EHHADH*, *FADS*), the urea cycle (*ARG2*, *ASL*, *ASS1*, *CPS1*), bile acid synthesis (*ACOX2*), and mineral absorption (*MT1A*, *MT1E*, *MT2A*). The expression of *DPP4* and *QRFPR*, genes associated with energy metabolism, was also negatively and positively affected, respectively.

### 2.4. GO Analysis of MUN-Associated Metabolic Pathways

Analysis of differentially expressed genes between the LN and HN groups revealed that MUN modulated the gene expression associated with hepatic metabolism. To understand the pathways in which the differentially expressed genes were enriched, gene ontology (GO) and Kyoto Encyclopedia of Genes and Genomes (KEGG) pathway analyses were performed using genes whose expression in the LN fetal liver was > 1.25-fold upregulated (1397 genes) or downregulated (1537 genes) when compared with those in the HN fetal liver (Table 4 and Table 5).

The downregulated genes in the LN fetal liver were significantly enriched in the following KEGG pathways: biosynthesis of antibiotics, glycolysis/gluconeogenesis, PPAR signaling pathway, peroxisome, protein digestion and absorption, tyrosine metabolism, metabolism of xenobiotics by cytochrome P450, and fatty acid degradation (*p* ≤ 0.001, Table 4). In addition, the downregulated genes were associated with GO terms (biological process) such as cholesterol biosynthetic process, fatty acid homeostasis, monocyte chemotaxis, cellular response to interferon-gamma, glucose homeostasis, chemokine-mediated signaling pathway, urea cycle, and neutrophil chemotaxis (*p* ≤ 0.001).

Meanwhile, the upregulated genes in the LN fetal liver were enriched in the following KEGG pathways: ribosome biogenesis in eukaryotes, RNA transport, aminoacyl-tRNA biosynthesis, protein processing in endoplasmic reticulum, HIF-1 signaling pathway, MAPK signaling pathway, and mineral absorption (*p* ≤ 0.05) (Table 5). The upregulated genes were also associated with the following GO terms (biological process): rRNA processing, protein folding, defense response to bacteria, chaperone-mediated protein folding requiring cofactors, ribosome biogenesis, RNA splicing, and negative regulation of growth (*p* ≤ 0.01). 

## 3. Discussion

### 3.1. Levels of Nutrients for Dams to Be Compared in the Present Study

Our study did not include animals that were fed 100% of their nutrient requirement. In a previous study investigating the effect of MUN on fetuses in female baboons, no significant differences in fetal body and liver weight were observed between dams fed on 70% and 100% of nutritional requirements during early-to-mid gestation period [23]. A similar result was obtained from a comparison between providing 85% and 140% of the required metabolic energy for cow feeding [24], suggesting that a relatively small restriction in maternal nutrition level may not lead to significant phenotypic effects on fetal carcasses. According to their results, a nutrient level within the range of 85 to 140% may not lead to significant differences in the fetal liver. Furthermore, a highly broad range of nutrient levels should be taken, employing the lowest level as much as possible. The 60% was considered as a minimum level to avoid accidents of pregnant cows [25]. During the last two months of gestation, Japanese Feeding Standard for Beef Cattle (JFSBC, 2008 ed.) recommends 125% and 141% levels of total TDN and CP, respectively, when the nutrient levels required for maintenance are considered as 100%, for pregnant Japanese Black cows [25,26]. Taking these previous studies into account, we considered that 120% of the requirement was an appropriate option as a counterpart treatment to the low level of 60%.

We considered it necessary to use an experimental design that caused a significant phenotypic alteration to understand the molecular basis underlying the phenotypic effect of MUN on fetal liver development, as discussed in our previous study [21]. Consequently, it was concluded that 120% of the requirement was an appropriate option as a counterpart treatment to the low level of 60%. Under these experimental conditions, a significant reduction in fetal liver mass was observed in the LN group [22]. This enabled us to further investigate the molecular mechanism underlying the effect of MUN on fetal liver growth in this study. Thus, we also focused on 60% and 120% of the nutrient requirement for comparison; however, 100% of the requirement might be the best nutritional standard level and is important to study as a control level to evaluate the nutritional effect, which might differ from cases using 60% and 120% levels.

### 3.2. Major Impacts of MUN on Fetal Liver

**Figure 5:**
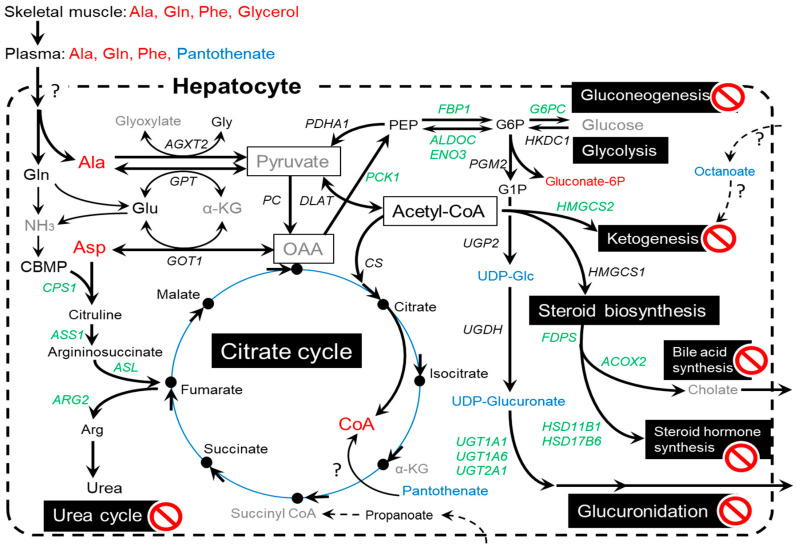
Hypothetic scheme of influences of maternal nutrient restriction on the fetal liver molecular pathways and metabolisms. Genes but not metabolites are italicized. Increased and decreased metabolites in the LN fetuses are indicated in red and blue, respectively. Green genes are downregulated in the LN fetuses. Grayed metabolites were not determined in this study.

In this study, MUN altered the metabolomic profile of the fetal liver. Among various maternal nutritional restriction conditions, we examined the effects of restriction of global nutrition including protein, fat, and total digestible nutrients (TDNs), during whole gestation. This nutrient-restricted condition markedly decreased BW and liver weight in fetuses, as well as the skeletal muscles, kidney, thymus, spleen, heart, lung, and rumen [22]. The liver plays a pivotal role not only in the biosynthesis of bile acid, cholesterol, and steroid hormones but also in the energy metabolism and excretion of NH_3_ and xenobiotics, which are essential for the homeostasis of systemic metabolism. Maternal nutrient restriction during gestation adversely affects liver size and metabolism in fetuses [2], the mechanism of which is not fully understood. In the current study, we observed alterations in various metabolite levels and gene expression in the liver of LN fetuses associated with AA metabolism, glycolysis/gluconeogenesis, ketogenesis, steroid biosynthesis, the urea cycle, mineral absorption, primary bile acid synthesis, and lipid metabolism (Figure 5). The levels of ATP and AMP and the AMP/ATP ratio were not significantly different between the LN and HN groups. This suggests that energy balance was maintained in the LN fetal liver to the same level as in the HN fetal liver at the expense of whole energy consumption in nutrient-restricted conditions.

### 3.3. Fetal Liver Metabolites and Metabolisms Affected by MUN

#### 3.3.1. Amino Acid Metabolism and Urea Cycle

Aspartate and alanine levels were significantly increased in the liver of LN fetuses; however, few studies have reported alterations in AA levels in the fetal liver of nutrient-restricted mothers during pregnancy, except for an example of arginine alteration in fetal rats [8]. Aspartate is associated with the urea cycle [27] (Table 3), arginine and proline metabolism, glutamate metabolism, tyrosine metabolism, and purine metabolism [28]. In the initiation step of urea synthesis, aspartate participates in the reaction with *ASS* to generate argininosuccinate as the donor of the amino group [29]. Aspartate can also be converted to oxaloacetate (OAA), which is further mobilized for energy production through the malate-aspartate shuttle. In the present study, aspartate accumulated in the liver of LN fetuses, indicating that the urea cycle was suppressed (Figure 5). This is consistent with the reduced expression of genes associated with the urea cycle, namely *CPS1*, *ASS1*, *ASL*, and *ARG2* (Figure 4) [30]. Suppression of the urea cycle is indicated by the results of the GO analysis (Table 4). Increased aspartate can be used as a precursor of the energy substrate for gluconeogenesis. However, in energy-restricted LN fetuses, as the expression of gluconeogenetic genes was reduced, aspartate could participate in the citrate cycle by being converted to OAA and being introduced into the malate-aspartate shuttle, rather than in gluconeogenesis or the urea cycle.

#### 3.3.2. Metabolisms Associated with Energy Production

Alanine, a major substrate of gluconeogenesis in the liver [31], was one of the AA increased in the LN group. Intriguingly, alanine was significantly increased in the *longissimus* muscle [21] and plasma (unpublished data) of LN fetuses. In LN fetuses, alanine increased in the muscle and could be transferred to the liver for energy production in the citrate cycle. However, as was the case with aspartate, the increased alanine was not likely to be utilized for gluconeogenesis because the gluconeogenic genes, *G6PC*, *FBP1*, and *PCK1* [32,33], were significantly suppressed in the LN group (Figure 4 and Figure 5). Genes encoding glycolytic enzymes, *ALDOC* and *ENO3*, were also downregulated, which could lead to an increase in gluconate-6P. These results were confirmed by the results of GO and KEGG pathway analyses showing that the downregulated genes were associated with glycolysis and gluconeogenesis (Table 4), which means that glycolysis/gluconeogenesis was suppressed in the liver of LN fetuses. 

Furthermore, *DPP4* was downregulated by MUN participate in glucose homeostasis. *DPP4* is associated with reduced insulin sensitivity and increased hepatic fat accumulation [34,35,36]. In lactating cows with subclinical ketosis, non-esterified fatty acids and triglycerides were decreased by intravenous injection of DPP4 [37]. Our results showed that *DPP4* expression was suppressed in the livers of the LN group. In addition, the system of QRFPR (also known as *GPR103*) and its peptide ligand RFa26 is involved in the regulation of food intake in the hypothalamus, as well as in glucose metabolism and energy metabolism [38], and it is thought to play a role in the development of obesity [38]. The RFa26/QRFPR system enhances the effect of insulin on glucose uptake in L6 myotubes [39] and induces fatty acid uptake and TAG accumulation in 3T3-L1 differentiated cells [40]. Intriguingly, MUN increased *QRFPR* expression in the livers of the LN group. Taken together, our results suggest that MUN affects liver glucose homeostasis and fatty acid metabolism through *DPP4* and *QRFPR* pathways in the fetuses of the LN group. 

The suppressed expression of gluconeogenetic genes *G6PC* and *PCK1* (*PEPCK*) was also observed in the fetal sheep livers of ewes fed a globally nutrient-restricted diet during gestation [41]. In contrast, in other studies examining fetuses of cows with protein restriction during the first trimester [16] or fetuses of goats with global nutrient restriction during 15 days in mid-gestation [42], expression of *G6PC*, *PCK1*, and/or glucose metabolism-related genes was activated in the liver of MUN fetuses compared with the control fetuses. The crucial ketogenic gene *HMGCS2* and genes associated with fatty acid metabolism were upregulated in the fetal liver of ewes fed a low-protein diet for 15 days in mid-gestation [20]. Together, these studies suggest that the activation or suppression of genes in MUN fetuses depends on the temporal and nutrient compositional conditions of maternal dietary restriction. Prolonged maternal restriction of global nutrients likely causes suppression of gluconeogenesis in the fetal liver. 

In contrast to genes involved in glycolysis/gluconeogenesis and energy substrate homeostasis, genes participating in the citrate cycle (e.g., *PC*, *CS*, *ACO1*, *MDH1*) were not downregulated in the LN group. Furthermore, ketogenesis was likely suppressed because of the reduced expression of *HMGCS2*, the key regulator initiating the ketogenic pathway [43] (Figure 4 and Figure 5). Collectively, these results suggest that energy substrates, such as AAs, were exclusively gathered in the citrate cycle, rather than glycolysis/gluconeogenesis, in the liver of the LN group. 

The citrate cycle was likely prioritized as the energy production pathway in the fetal liver of the LN group. Hepatic metabolism, including the urea cycle, steroid synthesis, and glucuronidation, was suppressed to save the compounds that can be mobilized for energy production, based on the reduced expression of genes involved in the metabolism of urea, steroids, and glucuronate in the LN group (Figure 5). The energy substrate precursors were thought to be metabolized less in the above-mentioned hepatic pathways and preferentially mobilized into the citrate cycle rather than gluconeogenesis for glucose supply to peripheral tissues. Acetyl-CoA is thought to be a key metabolite for providing resources for glycolysis and energy production (citrate cycle and ketogenesis), AA metabolism, fatty acid metabolism, steroid synthesis, and glucuronidation (Figure 5) [28]. The acetyl-CoA content did not differ between the LN and HN groups, possibly because of the altered distribution that made the limited amount of acetyl-CoA more for the citrate cycle and less for other pathways.

#### 3.3.3. Steroid Biosynthesis

MUN also impacts steroid biosynthesis, a vital metabolism specific to the liver [44], adrenal gland, and placenta in fetuses [45]. Cholesterol synthesis is controlled by *HMGCS1* that generates 3-hydroxy-3-methylglutaryl-CoA (HMG-CoA) [46] from acetoacetyl-CoA, which has crucial impacts on synthesis of steroid hormone, bile acid, vitamin D, and components involved in plasma lipoproteins and cell membrane [43]. In the liver of LN fetuses, *HMGCS1* expression was not suppressed (Figure 5), as in the case of gene expression of the rate-determining enzyme HMG-CoA reductase (*p* > 0.10). Meanwhile, the gene expression of *FDPS*, downstream of the mevalonate pathway in cholesterol synthesis, was suppressed. Moreover, the expression of *HSD11B1* and *HSD17B6*, the genes associated with steroid synthesis, were also suppressed. *HSD11B1* plays a major role in converting cortisone to cortisol, which reactivates circulating glucocorticoids and regulates fuel metabolism, energy partitioning, and body fat distribution in a tissue-specific manner [47,48]. The expression of *ACOX2*, a gene involved in primary bile acid synthesis, was also suppressed. These results indicate that MUN resulted in the suppression of steroid and primary bile acid synthesis in fetal liver metabolism, as indicated by MSEA and GO analysis (Table 3 and Table 4). Cholesterol synthesis could be also adversely affected. Suppressed steroid synthesis by MUN potentially leads to disruption of the plasma membrane and systemic hormonal regulation, which are essential for fetal development and growth. In sheep, 30% maternal nutrient restriction for 15 days in mid-gestation suppressed gene expression regulating steroid and cholesterol synthesis, including *HMGCS1*, in the fetal liver [20]. Meanwhile, global maternal nutrient restriction of 60% level in goats activated hepatic gene expression related to steroid biosynthesis and bile secretion, but not *HMGCS1* [49], and increased metabolites related to bile acid metabolism, such as taurochenodeoxycholate and taurocholate [42]. Taken together with the suppression of *FDPS*, *HSD11B1*, and *HSD17B6* observed in this study, it is likely that MUN disturbs the gene expression and metabolism associated with steroid and bile acid synthesis in the fetal liver. The regulated genes, metabolites, and up/downregulation likely depend on MUN conditions. As mentioned above, energy substrates such as acetyl-CoA might be mobilized into the citrate cycle at the expense of the suppression of steroid and bile acid biosynthesis.

#### 3.3.4. Glucuronidation for Catabolism of Steroid and Drugs

In the fetal liver, glucuronidation-associated gene expression (*UGT*s) and metabolites such as UDP-glucuronate were lowered by MUN. Glucuronidation is essential for drug metabolism and catabolism that are specific roles of the liver [50]. UDP-glucose is a key metabolite in the synthesis of glucuronate via UDP-glucuronate and is further converted to glucuronate by *UGT1A1*, *UGT1A6*, and/or *UGT2A1* for subsequent glucuronidation of waste metabolites [50]. Endogenous substrates for glucuronidation include bilirubin, bile acids, and steroids, whereas xenobiotics such as drugs and pollutants are detoxified by UGT enzymes [51]. In the liver of LN fetuses, UDP-glucose/UDP-galactose and UDP-glucuronate levels were decreased, and the expression of *UGT1A1*, *UGT1A6*, and *UGT2A1* were suppressed (Figure 5). These results indicate that MUN affects glucuronidation, which potentially leads to a deficiency in drug and steroid catabolism. Although the effect of MUN on fetal liver UGT activity has never been examined, the nutrient restriction has been shown to affect UGT activity in the livers of adult cows [52]. For example, alterations in nutritional level and high fat content can affect the expression of *UGT1A1* and *UGT1A6* in the liver, possibly through proliferator-activated receptor α [53]. The effect of MUN on the metabolism of sugars and nucleotide sugars was indicated in the MSEA results (Table 4), which could have resulted from lowered *UGT* levels in the disturbed glucuronate pathway in the LN group. As in the case of steroid synthesis, the energy substrates in glucose metabolism might be mobilized into the citrate cycle at the expense of glucuronidation for the metabolism of drugs and waste such as steroid hormones and bilirubin. 

#### 3.3.5. Lipid and Fatty Acid Metabolisms

In LN fetuses, gene expression associated with lipid and/or fatty acid metabolism (*ANGPTL4*, *APOA5*, *EHHADH*, *FADS2*) was suppressed, which was also indicated by the PPAR signaling pathway and fatty acid homeostasis/degradation in the GO and KEGG analyses (Table 4). *DPP4*, one of the genes suppressed by MUN, is involved in Western-diet-induced hepatic steatosis through hepatic triacylglycerol and diacylglycerol accumulation [35]. Although lipids and fatty acids were not sufficiently profiled by CE-TOFMS metabolomics, the transcriptomic results indicated that lipid and fatty acid metabolism were affected by MUN. In accordance with alterations in gene expression, MSEA results showed that MUN affected the metabolism of propanoate in the liver of the LN group (Table 3), in which 4-amino-3-HBA, 2-HBA, and octanoate were decreased (Table 2). Propanoate metabolism in the ruminant liver is crucial for glucose production in peripheral tissues [31], in which propanoate can be metabolized into propanoyl-CoA by propanoyl-CoA synthetase [54] and further introduced into the citrate cycle. 2-HBA can be mobilized into 2-oxobutanoate by lactate dehydrogenase, then into propionyl-CoA, and finally into the citrate cycle via succinyl-CoA (KEGG pathway: bta00640). Accordingly, in the LN group, the 1.25-fold increase in CoA (*p* = 0.094) and the decrease in 2-HBA compared with the HN group might be associated with propanoate metabolism.

It is also notable that *ANGPTL4* and *APOA5* play important roles as regulators of triglyceride metabolism in other tissues, including adipose tissue, through the circulation [55,56,57]. Intriguingly, the expression of the hepatokine *ANGPTL4* also decreased in the skeletal muscle of the LN group [57]. This suggests that MUN alters systemic lipid metabolism not only in the liver but also in skeletal muscle and adipose tissues through disruption of *ANGPTL4* and *APOA5* expression in the fetal liver and/or skeletal muscle. In addition, ANGPTL4 upregulates cholesterol synthesis in the liver secondary to inhibition of LPL- and HL-dependent hepatic cholesterol uptake [58]. Accordingly, decreased *ANGPTL4* expression might be associated with suppression of LN hepatic cholesterol synthesis, with which decreased *HSD11B1* expression could be associated [59].

#### 3.3.6. Other Metabolites and Gene Expression

In the liver of the LN group, ophthalmate accumulated more than two-fold compared to that in the HN group. Ophthalmate is generated through consecutive reactions with γ-glutamylcysteine synthetase and glutathione synthetase; therefore, it is thought to be a biomarker of oxidative stress caused by a lack of glutathione, especially from liver disorders [60]. Given that ophthalmate generally accumulates in tissues with low cysteine content, our results suggest that the cysteine level in the LN group was relatively low. The increase in glutathione derivatives (lactosylglutathione, methylglutathione) in the LN group (Figure 1) might also be a byproduct of glutathione shortage. 

In the LN group, there were fewer upregulated genes in the qPCR results than the downregulated genes. According to GO and KEGG analyses, most of the upregulated genes in the microarray analysis were associated with the cellular translational system and process (Table 5), although a few were confirmed by qPCR. The upregulated genes (*MT1A*, *MT1E*, and *MT2A*) were associated with mineral absorption and negative regulation of growth. Metallothioneins (MTs) comprise MT1-MT4 subgroups and are considered to have multiple roles in the storage of essential metals, capture of toxic heavy metals, and reduction of the toxic effects of free radicals [61]. MTs are induced not only by metals and hormones but also by oxidative stress. Under MUN conditions, the expression of *MT1A*, *MT1E*, and *MT2A* could be induced by a shortage of essential metals, or otherwise by oxidative stress, which is in line with the ophthalmate accumulation observed in the liver of LN fetuses. 

As indicated by the increased ophthalmate and MT gene expression, oxidative stress was induced in the liver of the LN group. In the fetal liver of ewes fed diets with restricted calories [11] or global nutrients [17], downregulated gene expression and decreased antioxidant activity were observed, which could lead to increased oxidative stress [17]. The liver of the LN group in the present study could also suffer from oxidative stress caused by a deficiency in energy supply. 

In the LN group, 2-AEP levels were decreased in the liver. However, the correlation between FGR and 2-AEP has not yet been elucidated. Rats and other higher vertebrates cannot generate 2-AEP, which contains a carbon-phosphorus bond [62,63]. Ruminant protozoa can synthesize protein-bound and lipid-bound forms of 2-AEP as well as the free form of 2-AEP [64]. Recently, we reported that 2-AEP decreased in the skeletal muscle of LN fetuses [21]. In the rat liver, 2-AEP is incorporated into phosphonolipids, such as diacylglyceryl-AEP [65], which suggests that 2-AEP is a membrane component. Thus, 2-AEP in the bovine fetus may be taken up from pregnant cows through the placenta and utilized as a membrane component. However, the mechanism by which 2-AEP is decreased in the liver and skeletal muscle of LN fetuses remains unknown. Further studies are needed to examine the biological role of dysregulated 2-AEP in the LN fetal liver. 

The liver of the LN group also showed a higher level of 3-APRP-1,2-diol than that of the HN group by 2.62-fold of the LN/HN ratio. 3-APRP-1,2-diol is a sphingophospholipid found in the bacterial fraction of sheep rumen contents [66] but has never been reported in vertebrate tissues. In contrast to 2-AEP, which was decreased in both skeletal muscle and liver of the LN group, 3-APRP-1,2-diol was increased in the liver but was not detected in the muscle of the LN group [21]. Although the reason that 3-APRP-1,2-diol was increased in the LN group is unclear, it is likely that it came from the rumen of the pregnant cows through the placenta and that it might be taken up into the liver as an extraneous compound to be excreted. 

## 4. Materials and Methods

### 4.1. Animals and Dietary Treatments

This study was performed on 11 multiparous Japanese Black cattle (initial BW 488 ± 9.6 kg) at the Iriki farm of Kagoshima University and the farm of the Western Region Agricultural Research Center, NARO. Animals were maintained according to the Guide for the Care and Use of Experimental Animals [67]. The experimental design was approved by the Animal Care and Use Committee of the Kagoshima University (#A18007). Animal management was performed as described previously [21]. Briefly, individual diets were designed for pregnant Japanese Black cows to meet 60% or 120% of the energy requirement and other nutrients based on the standard diet that was calculated for BW before pregnancy, according to the JFSBC (2008 ed.) [25]. The diet comprised formula feed, total mixed ration, and rice straw, as designed previously [22]. The recommended contents of dry matter (DM), neutral detergent fiber, acidic detergent fiber, ash, CP, Ca, and phosphorus in the diet were 68.0%, 56.1%, 36.0%, 11.1%, 8.0%, 0.6%, and 0.3% of DM, respectively. Cows were randomly assigned to the LN (*n* = 5) and HN (*n* = 6) diet groups and were fed their respective diets during gestation. The metabolizable energy of the mixture feed for the 100% requirement was estimated as 8.56 MJ/kg DM. Cows were subjected to artificial insemination (AI) using male-sorted semen from an identical sire. In total, 11 fetuses were obtained from cows through cesarean section at the Kagoshima University Veterinary Teaching Hospital.

### 4.2. Sample Collection 

The fetuses were euthanized by exsanguination at day 260 ± 8.3 of gestation, after injecting lidocaine (AstraZeneca, Osaka, Japan) into the jugular vein. Liver samples were collected from the right side of the dissected fetal carcass, frozen with liquid nitrogen or soaked in RNAlater^®^ (Thermo Fisher Scientific, Tokyo, Japan), and stored at −80 °C until used for subsequent analyses.

### 4.3. Sample Preparation for CE-TOFMS

Among the 11 fetuses, those with the lowest BW in the LN group (*n* = 4) and those with the highest BW in the HN group (*n* = 4) were selected. Liver samples were subjected to metabolomic analysis. Frozen liver pieces (53.4–90.9 mg) were immediately immersed in a solution containing 50% acetonitrile and 10 μM internal standard solution 1 (Human Metabolome Technologies, Tsuruoka, Japan) at 0 °C and homogenized twice at 1500 rpm for 120 s. The samples were centrifuged at 2300 g at 4 °C for 5 min. The upper layer solution was filtered through a Millipore 5-kDa cutoff membrane. The filtrate was lyophilized, suspended in Milli-Q water, and analyzed using CE-TOFMS.

### 4.4. CE-TOFMS and the Data Analysis

CE-TOFMS was performed using an Agilent capillary electrophoresis system equipped with an Agilent 6210 time-of-flight mass spectrometer, Agilent 1100 isocratic high-performance liquid chromatography pump, Agilent G1603A CE-MS adapter kit, and Agilent G1607A CE-ESI-MS sprayer kit (Agilent Technologies, Waldbronn, Germany). The analytical conditions were identical to those used in a previous study [21]. 

Raw data were processed using MasterHands, as described previously [21]. Briefly, among the detected compounds, those annotated in the Human Metabolome Database (ver. 4.0, http://www.hmdb.ca/, accessed on 27 January 2022) or KEGG database (http://www.genome.jp/kegg/, accessed on 27 January 2022) were further analyzed. The relative contents of the annotated compounds were determined by comparing the peaks of compounds with the same MS properties. To compare the relative content of the compounds between the LN and HN groups, the peak areas were normalized to those of the internal standards and sample weights. The abundance of each compound used for comparative analysis was set to zero when the level of the compound was not detected. The file conversion of raw MS data, peak picking, reduction of noise, and alignment of data for multiple samples was conducted as previously described [21].

### 4.5. RNA Preparation and Complementary DNA (cDNA) Synthesis

For microarray analysis, total RNA was extracted from liver samples using the mirVana^TM^ microRNA isolation kit (Thermo Fisher Scientific, Tokyo, Japan) following the manufacturer’s instructions. RNA quantity and quality were determined using an Agilent Bioanalyzer 2100 with an RNA 6000 Pico kit (Agilent Technologies, Santa Clara, CA, USA). For polymerase chain reaction (PCR) analysis, total RNA was prepared using ISOGEN II (Nippon Gene, Toyama, Japan). cDNA synthesis was performed from total RNA using ReverTra Ace qRT-PCR Master Mix (Toyobo), following the manufacturer’s instructions.

### 4.6. Microarray Analysis

The fetuses analyzed were those with the lowest BW in the LN group (*n* = 4) and those with the highest BW in the HN group (*n* = 4). Total RNA samples from four fetuses from the HN and LN groups were applied to a Bovine (v2) Gene Expression 4 × 44K Microarray (Agilent). The signals of the hybridized probes were detected using an Agilent microarray scanner (Agilent). The results were normalized by the quantile method using GeneSpring GX (Agilent). Array data were deposited in the National Center for Biotechnology Information (NCBI) Gene Expression Omnibus (GEO) database and can be accessed through the GEO Series accession number GSE191179 (http://www.ncbi.nlm.nih.gov/geo, accessed on 27 January 2022).

### 4.7. Quantitative Real-Time PCR (qRT-PCR) Analysis

Gene expression in the LN (*n* = 5) and HN (*n* = 6) groups was analyzed by qRT-PCR using a CFX96 thermal cycler (Bio-Rad, Hercules, CA, USA) with the QuantiTect SYBR Green PCR kit (Qiagen) and the primers are listed in Table S2. The ribosomal protein lateral stalk subunit P0 (*RPLP0*) was used as an internal control. Melting curve analysis was performed to confirm the specificity of amplification reactions.

### 4.8. Functional Annotation of Target Genes

The genes of interest were analyzed using the Database for Annotation, Visualization, and Integrated Discovery (version 6.7, http://david.abcc.ncifcrf.gov, accessed on 27 January 2022) with Bos taurus as the background species to enrich GO terms and characterize the KEGG pathway terms defined by KEGG (http://www.genome.jp/kegg/, accessed on 27 January 2022) for the respective biological processes associated with the effect of MUN. Several of the differentially expressed genes involved in the extracted terms were further tested by qRT-PCR. 

### 4.9. Statistical Analyses

The effects of nutrient levels on metabolites and gene expression were tested. The data were analyzed using the two-sided Student’s *t*-test for metabolomics results or the one-sided Student’s *t*-test for PCR results, based on the trend of gene expression in microarray analysis. Differences were considered significant at *p* ≤ 0.05, or a trend at *p* ≤ 0.10. HCA and MSEA, using KEGG as a metabolite set library, were performed using MetaboAnalyst 5.0 (https://www.metaboanalyst.ca/MetaboAnalyst/faces/home.xhtml, accessed on 27 January 2022).

## 5. Conclusions

This study examined the effect of whole gestational MUN on fetal liver growth at the late gestational stage and revealed alterations in the expression of genes associated with glucose homeostasis, glucuronidation, steroid synthesis, and the urea cycle in the liver of LN fetuses. The levels of metabolites relevant to glucuronidation, such as UDP-glucuronate and UDP-glucose/UDP-galactose, were also decreased in the LN group. The downregulated glucogenic and ketogenic genes in the LN group suggest that glucose and ketone bodies were not generated in the liver for delivery to peripheral tissues, such as skeletal muscle and adipose tissue. Thus, it is likely that the liver of LN fetuses prioritizes energy saving for survival under MUN conditions at the expense of maintenance of energy homeostasis, hormonal regulation, nitrogen excretion, and waste metabolism. 

## Figures and Tables

**Table 1:** Phenotypic effect of maternal nutrient restriction on fetal liver.

	LN (*n* = 6)		HN (*n* = 5)		
	Mean *	SE *	Mean	SE	*p*-Value
Age (d)	260.7	1.6	261.6	1.5	0.684
BW (kg)	23.4	2.2	32.5	0.5	0.005
Liver (g)	487.3	41.8	627.6	19.8	0.020
%BW	2.1	0.07	1.93	0.03	0.071

* The values of the LN group were reported previously [21,22].

**Table 2:** Top 20 differently expressed metabolites in fetal liver between LN and HN groups *.

Compound	LN (*n* = 4)		HN (*n* = 4)		Ratio (LN/HN)	*p*-Value
	Mean	SE	Mean	SE		
Aspartate	16.500	0.559	11.750	0.545	1.40	0.002
4-Amino-3-hydroxybutyrate	0.009	0.001	0.017	0.001	0.55	0.012
2-Aminoethylphosphonate	0.043	0.005	0.097	0.013	0.44	0.013
Betaine aldehyde	0.088	0.008	0.045	0.007	1.95	0.014
UDP-glucose/UDP-galactose	0.133	0.014	0.225	0.019	0.59	0.015
*N*^5^-Ethylglutamine	0.320	0.022	0.558	0.057	0.57	0.015
UDP-glucuronate	0.248	0.017	0.335	0.016	0.74	0.019
2-Hydroxybutyrate	0.061	0.021	0.163	0.022	0.38	0.028
Glycerol	7.925	0.828	4.925	0.399	1.61	0.030
3-Aminopropane-1,2-diol	0.017	0.001	0.007	0.003	2.62	0.032
Alanine	31.750	1.431	26.500	0.829	1.20	0.033
Octanoate	0.002	0.000	0.013	0.003	0.19	0.035
6-Phosphogluconate	0.173	0.017	0.102	0.017	1.70	0.044
Gly-Leu	0.021	0.001	0.029	0.002	0.72	0.046
Ophthalmate	14.250	1.244	6.875	2.280	2.07	0.049
Pantothenate	0.170	0.009	0.230	0.022	0.74	0.071
*N*^6^,*N*^6^,*N*^6^-Trimethyllysine	0.130	0.009	0.103	0.006	1.26	0.084
γ-Glu-Ser	0.072	0.006	0.049	0.008	1.48	0.085
Methionine sulfoxide	0.007	0.003	0.019	0.005	0.36	0.091
Gluconate	0.308	0.020	0.400	0.035	0.77	0.092

* Values in table are relative content levels (arbitrary unit). LN and HN: Low and high nutrition treatment, respectively.

**Table 3:** Top 20 fetal liver metabolisms different between LN and HN fetuses *.

Metabolism/Pathway	Hits/Total Metabolites	*p*-Value	Increased in LN	Decreased in LN
Urea Cycle	2/29	0.0013	Asp, Ala	
Starch and Sucrose Metabolism	3/31	0.0014		Glucuronate, UDP-Glc, UDP-glucuronate
Malate-Aspartate Shuttle	1/10	0.0019	Asp	
Nucleotide Sugars Metabolism	3/20	0.0024		UDP-Gal, UDP-Glc, UDP-glucuronate
Arginine and Proline Metabolism	3/53	0.0034	Asp	FAD, Phosphocreatine
Propanoate Metabolism	3/42	0.0035	CoA	FAD, 2-HBA
Glutamate Metabolism	5/49	0.0036	Asp, Ala, CoA	FAD, GSSG
Beta-Alanine Metabolism	5/34	0.0050	Asp, His, CoA	FAD, Pantothenate
Tyrosine Metabolism	2/72	0.0065	Asp	FAD
Purine Metabolism	2/74	0.0065	Asp	FAD
Aspartate Metabolism	2/35	0.0065	Asp	FAD
Porphyrin Metabolism	2/40	0.0070		FAD, UDP-glucuronate
Galactose Metabolism	3/38	0.0072	Glycerol	UDP-Gal, UDP-Glc
Betaine Metabolism	4/21	0.0076	BTL	Dimethylglycine, FAD, Met
Glycerolipid Metabolism	3/25	0.0095	CoA, Glycerol	FAD
Warburg Effect	3/58	0.0116	6-Phosphogluconate, CoA	FAD
Pantothenate and CoA Biosynthesis	3/21	0.0138	CMP, CoA	Pantothenate
Lactose Synthesis	2/20	0.0148		UDP-Gal, UDP-Glc
Sphingolipid Metabolism	2/40	0.0170	Ser	UDP-Glc
Androgen and Estrogen Metabolism	1/33	0.0186		UDP-glucuronate

* Metabolisms and pathways that were extracted in MSEA using top 50 different metabolites are listed above. BTL; betaine aldehyde, CMP; cytidine monophosphate, CoA; coenzyme A, GSSG; oxidized glutathione, 2-HBA; 2-hydroxybutyrate, FAD; flavin adenine dinucleotide, UDP-glucuronate; uridine 5′-diphosphate-glucuronate, UDP-Glc; UDP-glucose, UDP-Gal; UDP-galactose. Amino acids and peptides are shown in three letter code.

**Table 4:** Altered fetal liver metabolisms and pathways extracted from downregulated genes *.

Category	Term	*p*-Value	Fold Enrichment	Differentially Expressed Genes **
**KEGG Pathway**				
	bta01100:Metabolic pathways	<0.001	1.698	*ENO3*, *G6PC*, *PIPOX*, *HSD11B1*, *ADH4*, *ASL*, *PCK1*, *FDPS*, *CPS1*, *TAT*, *HMGCS2*, *UGT2A1*, *ARG2*, *UGT1A1*, *ACOX2*, *EHHADH*, *ALDOC*, *FBP1*, *SAO*, *HSD17B6*, *UGT1A6*, *ASS1*
	bta01130:Biosynthesis of antibiotics	<0.001	2.647	*TAT*, *ENO3*, *ASL*, *HMGCS2*, *PCK1*, *FDPS*, *ARG2*, *ASS1*, *EHHADH*, *ALDOC*, *FBP1*
	bta00010:Glycolysis/Gluconeogenesis	<0.001	3.463	*G6PC*, *ENO3*, *ADH4*, *ALDOC*, *PCK1*, *FBP1*
	bta05204:Chemical carcinogenesis	<0.001	3.289	*UGT1A1*, *HSD11B1*, *ADH4*, *UGT2A1*, *UGT1A6*
	bta03320:PPAR signaling pathway	<0.001	3.289	*APOA5*, *FADS2*, *ACOX2*, *EHHADH*, *ANGPTL4*, *PCK1*
	bta04146:Peroxisome	<0.001	2.885	*PIPOX*, *ACOX2*, *EHHADH*
	bta04974:Protein digestion and absorption	<0.001	2.885	*PRCP*, *DPP4*, *COL1A1*, *COL1A2*,
	bta00350:Tyrosine metabolism	<0.001	3.939	*SAO*, *ADH4*, *TAT*
	bta00980:Metabolism of xenobiotics by cytochrome P450	<0.001	3.286	*UGT1A1*, *HSD11B1*, *ADH4*, *UGT2A1*, *UGT1A6*
	bta00071:Fatty acid degradation	<0.001	3.843	*ADH4*, *EHHADH*
**GO: Biological Process**				
	GO:0006695~cholesterol biosynthetic process	<0.001	7.442	*FDPS*, *APOA5*, *HMGCS2*
	GO:0006958~complement activation, classical pathway	<0.001	5.953	
	GO:0055089~fatty acid homeostasis	<0.001	7.938	
	GO:0002548~monocyte chemotaxis	<0.001	4.106	*CCL14*, *CCL21*, *CCL5*
	GO:0071346~cellular response to interferon-gamma	<0.001	3.638	*CCL14*, *CCL21*, *CCL5*
	GO:0055114~oxidation-reduction process	<0.001	1.696	*SAO*, *HSD17B6*, *FADS2*, *PIPOX*,
	GO:0042593~glucose homeostasis	<0.001	2.492	*G6PC*, *PRCP*
	GO:0070098~chemokine-mediated signaling pathway	<0.001	3.247	*CCL14*, *CCL21*, *CCL5*
	GO:0000050~urea cycle	0.001	8.505	*ARG2*, *CPS1*, *ASL*, *ASS1*
	GO:0030593~neutrophil chemotaxis	0.001	3.040	*CCL14*, *CCL21*, *CCL5*

* Top 10 GO metabolisms and KEGG pathways extracted at *p* < 0.05 are listed. *PIPOX*; pipecolic acid oxidase, *ADH4*; alcohol dehydrogenase 4 (class II), pi polypeptide, *TAT*; tyrosine aminotransferase, *SAO*; amine oxidase, copper containing 3, *PRCP*; prolylcarboxypeptidase, *COL1A1*; collagen type I alpha 1, *COL1A2*; collagen type I alpha 2, *CCL14*; chemokine (C-C motif) ligand 14, *CCL21*; chemokine (C-C motif) ligand 21, *CCL5*; chemokine (C-C motif) ligand 5. ** Differential expression of the listed genes was validated by qPCR (*p* ≤ 0.10).

**Table 5:** Altered fetal liver metabolisms and pathways extracted from upregulated genes *.

Category	Term	*p*-Value	Fold Enrichment	Differentially Expressed Genes **
**KEGG Pathway**				
	bta03008:Ribosome biogenesis in eukaryotes	<0.001	4.8155	
	bta03013:RNA transport	<0.001	2.3926	
	bta00970:Aminoacyl-tRNA biosynthesis	<0.001	3.9018	
	bta05230:Central carbon metabolism in cancer	<0.001	3.0680	*FGFR2*
	bta04141:Protein processing in endoplasmic reticulum	0.001	2.0779	
	bta04066:HIF-1 signaling pathway	0.005	2.2862	
	bta05200:Pathways in cancer	0.013	1.4705	*FGFR2*
	bta05031:Amphetamine addiction	0.015	2.4022	*PPP1R1B*, *ATF4*
	bta04010:MAPK signaling pathway	0.025	1.5492	*FGFR2*, *ATF4*
	bta04978:Mineral absorption	0.028	2.6603	*MT2A*, *MT1A*, *MT1E*
**GO: Biological Process**				
	GO:0006364~rRNA processing	<0.001	4.6384	*RRP9*
	GO:0006457~protein folding	<0.001	*2.9962*	
	GO:0000462~maturation of SSU-rRNA from tricistronic rRNA transcript (SSU-rRNA, 5.8S rRNA, LSU-rRNA)	<0.001	5.0379	
	GO:0042742~defense response to bacterium	<0.001	2.8497	*CATHL4*, *CATHL3*
	GO:0008542~visual learning	0.001	3.6639	*PPP1R1B*
	GO:0051085~chaperone mediated protein folding requiring cofactor	0.002	8.0148	
	GO:0042254~ribosome biogenesis	0.002	4.1036	
	GO:0008380~RNA splicing	0.004	2.5647	
	GO:0045926~negative regulation of growth	0.005	6.4119	*MT2A*, *MT1A*
	GO:0000470~maturation of LSU-rRNA	0.006	4.8089	

* Top 10 GO metabolisms and KEGG pathways extracted at *p* < 0.05 are listed. *FGFR2*; fibroblast growth factor receptor 2, *PPP1R1B*; protein phosphatase 1 regulatory inhibitor subunit 1B, *ATF4*; activating transcription factor 4, *RRP9*; ribosomal RNA processing 9, small subunit (SSU) processome component, homolog (yeast), *CATHL3*; cathelicidin antimicrobial peptide. ** Differential expression of the listed genes was validated by qPCR (*p* < 0.10).

## Data Availability

Array data were deposited in the National Center for Biotechnology Information (NCBI) Gene Expression Omnibus (GEO) database and are accessible through GEO Series accession number GSE191179 (http://www.ncbi.nlm.nih.gov/geo).

## Supplementary Materials

The following are available online at https://www.mdpi.com/article/10.3390/metabo12030203/s1, Table S1: Relative levels of detected metabolites in LN and HN fetal liver by CE-TOFMS metabolomics, Table S2: PCR primers used in this study.
